# Unravelling the interplay: brain regional atrophy and neuropsychological function in early Alzheimer’s disease

**DOI:** 10.3389/fnagi.2025.1508849

**Published:** 2025-05-14

**Authors:** Doyun Heo, Min-Seong Kim, Yun-Jin Lee, Seon-Kyeong Kim, Yong Sung Kim, Wonjae Sung, Hee-Jin Kim

**Affiliations:** ^1^College of Medicine, Hanyang University Hospital, Seoul, Republic of Korea; ^2^Department of Medical and Digital Engineering, Hanyang University, Seongdong-gu, Seoul, Republic of Korea; ^3^Department of Neurology, College of Medicine, Hanyang University Hospital, Seoul, Republic of Korea

**Keywords:** Alzheimer’s disease, brain volumetry, cognitive function, correlation studies, retrospective studies

## Abstract

**Objectives:**

The structural changes in the brain differ between normal aging and Alzheimer’s dementia (AD). The results of cognitive function tests reflect structural changes in the brain in AD. This study aimed to determine the specific relationship between regional brain volume and neuropsychological subtest scores.

**Methods:**

Ninety-three patients with definitive diagnosis of AD (confirmed by PET) were retrospectively enrolled. An automated program Quick Brain Volumetry (QBraVo) was used to measure the regional gray matter (GM) volume of the participants. Each score of the Seoul Neuropsychological Screening Battery (SNSB) subset test was statistically analyzed to observe correlations between regional brain volumes and cognitive function. Results of the SNSB subset test were compared to the degree of brain volume atrophy.

**Results:**

The Controlled Oral Word Association Test (COWAT), Trail making test for the elderly (TMT–e), and Korean version Boston naming test (K-BNT) were strongly correlated with GM volume atrophy, mainly in the temporal lobe. Memory functions, including Seoul verbal learning test (SVLT), Rey complex figure test (RCFT) recall and recognition tests, were significantly correlated with both the temporal and frontal regions. Various tests reflecting frontal and executive functions did not reveal significant correlations with the frontal regions. The BNT test scores reflecting language function did not correlate with frontal atrophy. Tests reflecting visuospatial capability (RCFT) were also related to inferior frontal and temporal atrophies.

**Conclusion:**

In patients with AD, the results of most cognitive function tests are related to the degree of atrophy of the temporal and frontal cortices. Further research is necessary to determine the extent to which cognitive function test results are associated with brain atrophy.

## 1 Introduction

Alzheimer’s disease (AD) is the most prevalent type of dementia in middle-aged and older persons and is a gradual, irreversible, and degenerative brain disorder that affects memory, thinking, and behavior (Ferrarini et al., 2006). In AD progression, brain atrophy is an important clinical characteristic with Aβ pathology (Braak et al., 1999). There have been several studies examining regional brain volume and cognitive function. Some regional gray matter (GM) atrophy such as hippocampal volume related to memory impairment and frontal lobe injury linked to executive dysfunction is known to be associated with cognition (Bozzali et al., 2011). Specifically, medial temporal lobe (MTL) atrophy plays a central role in declarative memory (Pantel et al., 2004). Clinically, studies examining AD patients have suggested that memory is correlated to the temporal and frontal gyri, and executive function is correlated to the frontal region during the progression of AD (Duarte et al., 2006; Striepens et al., 2010; Cajanus et al., 2019).

GM atrophy begins at age 20 and persists, with frontal lobe atrophy occurring the fastest and temporal lobe atrophy occurring relatively slowly (Harada et al., 2013). This is in contrast to the characteristics of AD where severe temporal lobe atrophy has been observed to occur in the early stage (Farrow et al., 2007). Language ability and visuospatial function do not dramatically change during normal aging, and it is established that language ability remains constant until age 70 (Harada et al., 2013). Executive function is heavily influenced by age, and this appears to be related to the observation that frontal lobe atrophy occurs rapidly (Lacreuse et al., 2020). However, there was no correlation between brain volume and any neuropsychological (NP) test scores in healthy controls in some studies (Basso et al., 2006; Chee et al., 2009). In summary, brain volume-cognitive function relationships in the healthy group were different compared to those of the AD group, and a more pronounced correlation was observed in AD patients.

So far, studies analyzing these correlations specifically in patients with pathologically confirmed AD based on FBB PET are limited.

Therefore, our study aimed to fill this gap by focusing on AD patients with confirmed amyloid positivity to investigate the subtle relationship between cognitive performance, as indicated by neuropsychological testing, and brain volume changes during the early stages of AD. From this retrospective study examining brain volume and cognition, we discovered a new finding regarding the relationship between regional GM volume and cognitive function as represented by comprehensive NP test scores. First, we organized the demographic data of the patients with AD using magnetic resonance imaging (MRI) scans and comprehensive NP test scores. Second, we observed a correlation between comprehensive NP test scores and detailed brain volumes.

## 2 Materials and methods

### 2.1 Patients

**Figure 1:**
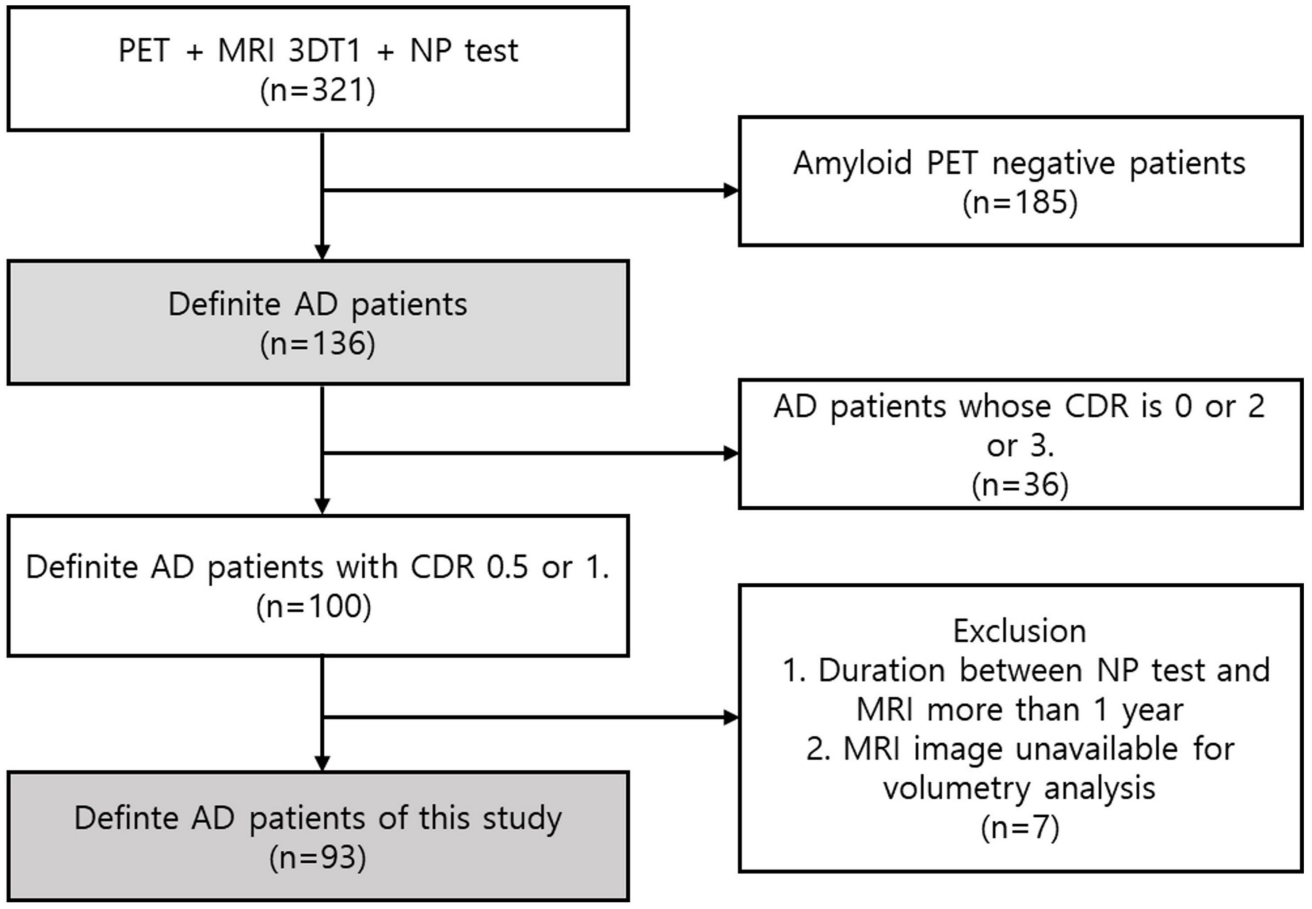
Participant enrolment. A total of 321 patients with PET, MRI, and comprehensive cognitive test results were selected from the historical patient records of the Neurology Department at a tertiary university hospital. Based on the NIA-AA research design, we chose 136 patients who had been diagnosed with dementia and Alzheimer’s pathologic changes. Next, we eliminated 100 patients with CDR scores of 1 or 0.5. Given that this study was performed retrospectively, the time between the MRI scan and the NP test for the selected patients could not exceed one year. With the exception of situations in which there was a year or more between the MRI and the NP test or in which the MRI pictures could not be analysed, we ultimately selected 93 research participants.

Among patients with past medical records from the neurology department at a tertiary university hospital, 321 patients with positron emission tomography (PET), MRI scans, and comprehensive neuropsychological test scores were filtered. We selected 136 patients who were diagnosed with Alzheimer’s pathologic changes with dementia based on the National Institute of Aging and the Alzheimer’s Association (NIA-AA) research framework (Jack et al., 2018). Patients with vascular injury were excluded to eliminate vascular contribution to cognitive impairment. Additionally, as part of the diagnostic process, these patients underwent dementia workup, including laboratory tests for thyroid function, vitamin B12 levels, syphilis serology, renal and liver function tests, common blood count (CBC), etc. Only those without any secondary causes of cognitive impairment were included in the study. Then, we filtered 100 patients whose Clinical Dementia Rating (CDR) scores were 0.5 (questionable impairment/dementia) or 1 (mild impairment) (Morris, 1993). Patients with CDR score of 0 (no dementia) were not included due to insufficient participant numbers meeting the criteria. Patients with CDR scores of 2 (moderate impairment) or 3 (severe impairment) were considered unsuitable for analyzing the correlation between brain atrophy and early subtle cognitive changes. For the selected participants, the time periods between the MRI scan and NP test were not greater than 1 year in consideration of the retrospective nature of this study. Therefore, we finally selected 93 study participants after excluding cases where the interval between the MRI and the NP test was longer than 1 year or where MRI images were not available for analysis. The mean interval between the MRI and neuropsychological assessments was 0.76 months (22.7 days), with a standard deviation of 1.71 months (51.4 days). The median interval was 0.1 month (3 days). From these 93 patients with confirmed AD based on FBB PET, we assessed the score data of the NP tests and MRI T1 images (Figure 1).

### 2.2 Neuropsychological assessment

Neuropsychological tests are frequently utilized on AD patients to assess cognitive function by determining the existence and severity of dementia, to make differential diagnoses, to track the course of the disease, and to track the effects of medications (Ahn et al., 2010). We used a complex and comprehensive neuropsychological diagnostic tool that is recognized in Korea as an effective test battery that can provide an in-depth and comprehensive evaluation of cognitive function (Ryu and Yang, 2023; Shin, 2010). The 29 subtests that comprise the cognitive function test can be categorized into 5 cognitive domains that include attention, language and related functions, visuospatial functions, memory, and frontal/executive functions (Ryu and Yang, 2023).

### 2.3 Magnetic resonance imaging analysis

Image acquisition MRI was performed using a 3.0-T Achieva system (Phillips, Best, The Netherlands) equipped with a standard quadrature head coil. The imaging data were collected with T1-weighted (T1W) three-dimensional (3D) MR images. We used the Quick Brain Volumetry (QBraVo) program, automated volumetric software based on the 8th version of the Statistical Parametric Map (SPM8) package (Wellcome Trust Centre for Human Neuroimaging, London, UK; http://www.fl.ion.ucl.ac.uk/spm), and MATLAB (The Mathworks, Inc., Natick, MA, United States) to measure the regional volume of GM, WM, and CSF from MR images (Ryu et al., 2022). QBraVo measures the volume divided into 53 areas that include 6 frontal (orbital, anterior, anterior medial, dorsolateral, inferior, and posterior medial), 3 temporal (anterior, medial, and lateral), and 2 parietal (medial and lateral) areas as well as occipital, central, cerebellar, and ventricular areas according to neuroanatomical boundaries (Ryu et al., 2022).

### 2.4 Statistical analysis

Descriptive statistics were used for the mean analysis of age, sex, years of education, Seoul Neuropsychological Screening Battery (SNSB) subtest scores, and regional GM volume. SNSB subtest scores were presented as standardized scores. We analyzed the regional brain volumes by using the ratio of each region to the Total Intracranial Volume (TIV). A Wilcoxon rank sum test was used to determine the significance of differences. Partial Correlation analysis was used to determine the correlation coefficients between regional brain volume and SNSB subtest raw scores after adjusting for age, sex, and years of education as confounding factors. We corrected the regional brain volume by dividing the TIV to adjust for developmental factors such as head size.

Descriptive statistics were conducted using R Statistical Software (version 4.4.2; R Core Team R, 2024). SPSS version 28.0 (IBM Corp., Armonk, NY, United States) was used for statistical analysis. Mechanical significance was set at *p* < 0.05*, *p* < 0.005**, and *p* < 0.001***.

## 3 Results

### 3.1 Demographic data

Ninety-three patients with AD (including 37 men and 56 women) were enrolled in this study. Participants were divided into two groups with CDR 0.5 (*n* = 67) and 1 (*n* = 26) (Table 1).

**Table 1:** Demographic data, independent sample t-test.

Variables		CDR 0.5 (*n* = 67)		CDR 1 (*n* = 26)		*p*-value
		Mean	SD	Mean	SD	
	Age	73	9	74	9	NS*
	Number of men/women	27/40		10/16		NS*
	Education year	11.2	4.3	9.3	6.7	NS*
Neuropsychological parameters						
Attention	Digit span forward	0.79	1.04	0.89	1.13	NS*
	Digit span backward	−0.22	0.86	−0.53	0.85	NS*
Language	Naming K-BNT	−0.85	2.01	−2.80	4.69	NS*
	Naming S-K-BNT	−0.54	0.98	−1.41	1.80	0.029**
Visuospatial functions	RCFT copy	−0.49	2.36	−2.79	5.77	0.007*
Frontal/executive functions	COWAT_semantic	−0.71	0.91	−1.19	1.01	0.035**
	COWAT_phonemic	−0.37	0.98	−0.81	1.10	NS
	Stroop test color reading	−1.00	1.54	−2.08	2.14	0.027**
	DSC	−0.26	1.01	−0.92	1.15	0.009*
	TMT_E_B	−0.59	1.94	−2.09	4.55	0.037***
Learning and Memory	SVLT immediate recall	−0.98	0.97	−1.86	1.38	0.004***
	SVLT delayed recall	−1.46	1.09	−2.19	0.96	0.005*
	SVLT recognition	−1.17	1.48	−2.49	1.99	0.004*
	RCFT immediate recall	−0.91	1.02	−1.71	0.94	0.003*
	RCFT delayed recall	−1.03	1.07	−1.94	1.03	0.001*
	RCFT Recognition	−0.84	1.49	−1.63	1.49	0.016*
Brain regional volumes						
Frontal regions	Orbitofrontal	0.70	0.10	0.63	0.09	<0.001
	(Lt & Rt)	0.75	0.12	0.68	0.08	<0.001
	Frontal anterior	0.95	0.11	0.93	0.08	NS
	(Lt & Rt)	1.07	0.10	1.05	0.07	NS
	Frontal anterior medial	1.42	0.09	1.41	0.09	NS
	(Lt & Rt)	1.42	0.09	1.42	0.15	NS
	Frontal dorsolateral	1.10	0.11	1.10	0.16	NS
	(Lt & Rt)	1.21	0.12	1.20	0.10	NS
	Frontal inferior	0.95	0.10	0.96	0.10	NS
	(Lt & Rt)	1.06	0.10	1.06	0.11	NS
	Frontal posterior medial	0.96	0.10	0.94	0.10	NS
	(Lt & Rt)	0.90	0.07	0.89	0.09	NS
Temporal regions	Temporal anterior	0.69	0.09	0.62	0.12	0.010
	(Lt & Rt)	0.77	0.09	0.73	0.11	NS
	Temporal medial	0.85	0.08	0.80	0.10	NS
	(Lt & Rt)	0.83	0.07	0.79	0.07	0.031
	Temporal lateral	2.52	0.21	2.43	0.23	NS
	(Lt & Rt)	2.64	0.21	2.60	0.26	NS
	Entorhinal cortex	0.15	0.02	0.14	0.02	NS
	(Lt & Rt)	0.14	0.02	0.13	0.02	0.040
	Hippocampus	0.22	0.03	0.19	0.04	0.006
	(Lt & Rt)	0.22	0.02	0.2	0.03	0.010
	TIV	1,296	124	1,270	107	NS

*p < 0.05, **p < 0.005, ***p < 0.001. Neuropsychological parameters are presented as standardized z-scores. Regional brain volumes are expressed as percentages, calculated by dividing the volume of each region by the Total Intracranial Volume (TIV). Lt: Left orbitofrontal lobe; Rt: Right orbitofrontal lobe.

We compared the mean age, years of education, neuropsychological parameters, and regional brain volume between the two groups. There was no significant difference in the attention subdomain assessed by the Digit Span test, while S-K-BNT (Short Korean version Boston naming test) (*p* = 0.029), which assesses the language function, showed significant differences. The RCFT copy (*p* = 0.007), which assesses visual function, also showed a significant difference between the two groups. In terms of parameters detailing frontal/executive functions, significant differences between the two groups were observed in the COWAT (Controlled Oral Word Association Test) semantic score (*p* = 0.035), Stroop test (*p* = 0.009), DSC (Digit symbol coding) (*p* = 0.009) and TMT-e (Trail making test for elderly) (*p* = 0.037). Additionally, differences between the two groups were observed for learning and memory fields as indicated by the SVLT (Seoul verbal learning test) immediate recall (*p* = 0.004), SVLT delayed recall (*p* = 0.005), SVLT recognition (*p* = 0.004), RCFT (Rey complex figure test) immediate recall (*p* = 0.003), RCFT delayed recall (*p* = 0.001), and RCFT recognition (*p* = 0.016).

Certain frontal brain regions differed between the two groups. Both the left (*p* < 0.001) and right orbitofrontal regions (*p* < 0.001) showed significant differences among frontal regions. For the temporal regions, left temporal anterior region (*p* = 0.010), right temporal medial region (*p* = 0.031), right entorhinal cortex (*p* = 0.040) and both the left (*p* = 0.006) and right hippocampus (*p* = 0.010) exhibited significant differences (Table 1).

### 3.2 Frontal/executive functions exhibited correlations mainly with temporal lobe

Correlation analyses between neuropsychological parameters and regional brain volumes were conducted using the combined sample of patients with CDR 0.5 and 1. This approach was chosen to avoid reducing the sample size and to maintain statistical power, while exploring associations in early-stage AD, as all participants were amyloid-positive. For comparison, subgroup analyses stratified by CDR level were also performed and are presented in Supplementary Tables 1–4.

We analyzed the correlations between frontal/executive function test scores and brain regions. Frontal function tests showed numerous significant correlations, primarily with temporal regions. The COWAT and TMT-e (TMT for the elderly) exhibited many significant correlations, while fewer correlations were observed in the Stroop and DSC tests with the temporal regions. However, there was not a great deal of correlation between frontal function and the frontal region itself. The number of regions showing significant correlations with the COWAT was the highest among the four frontal/executive function tests, with three regions exhibiting associations. The frontal inferior region that was correlated with the COWAT (r = 0.216, *p* < 0.05), DSC (r = 0.239, *p* < 0.05) and TMT (Trail making test) (r = −0.253, *p* < 0.05) was the most correlated among the six frontal regions.

**Figure 2:**
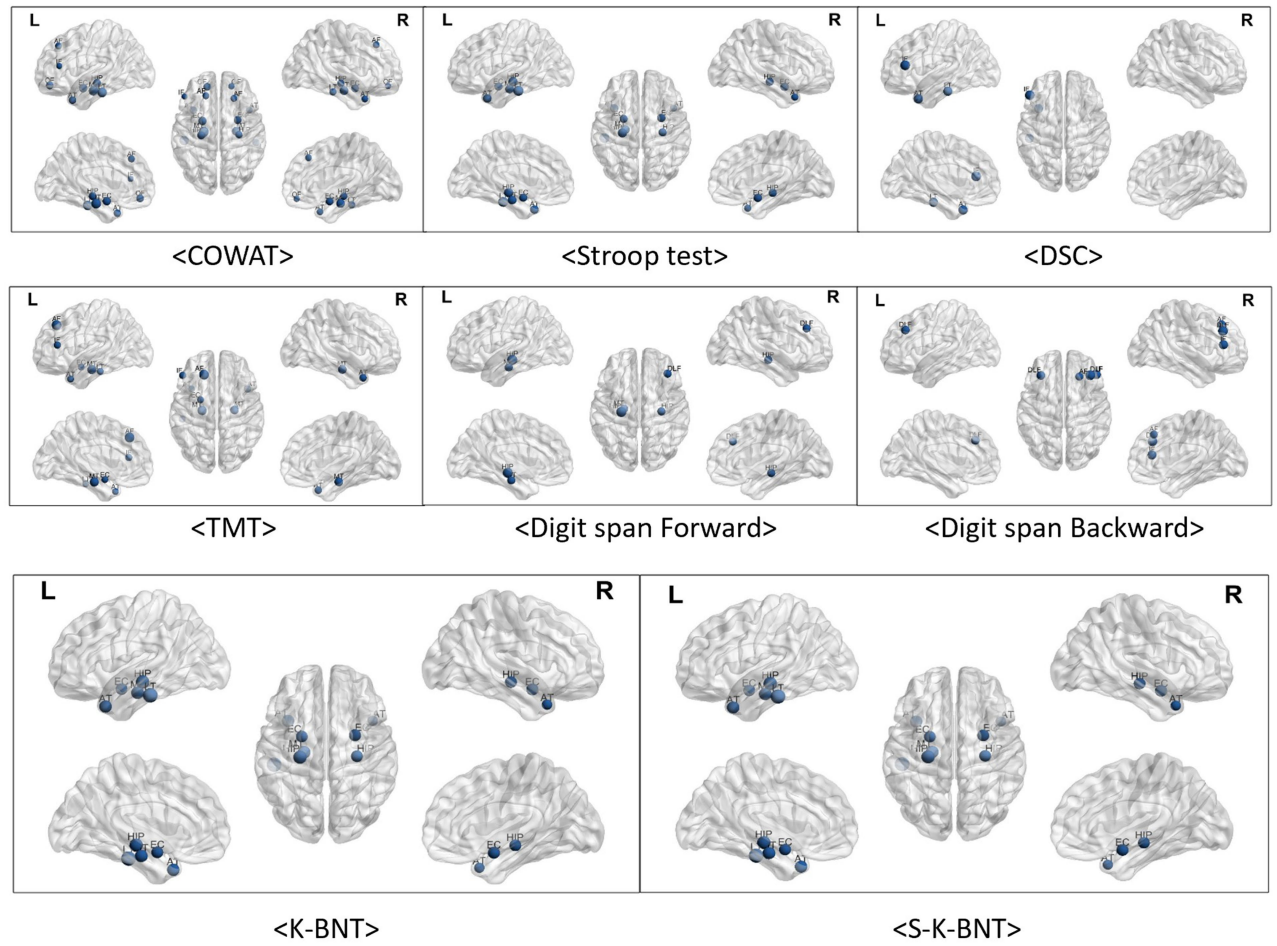
Visualized correlations between regional brain volume and frontal function. The frontal areas and functions did not significantly correlate. In particular, there was no relationship observed between frontal regions and K-BNT scores. Of the six frontal regions, the frontal inferior region exhibited the highest relationships with COWAT (r = 0.216, *p* < 0.05), DSC (r = 0.239, *p* < 0.05), TMT (r = −0.253, *p* < 0.05), and Digit span test (r = 0.261, *p* < 0.05). Numerous relationships were observed among temporal regions for COWAT, TMT-e, and K-BNT. Less associations between temporal areas and the Stroop, DSC, and Digit span tests were observed.

Tests assessing attention, digit span tests, showed a consistent correlation with the regional volumes of both the temporal and frontal lobes. Language function test results including K-BNT (Korean version Boston naming test) and S-K-BNT were not correlated with any of frontal regional volumes. However, K-BNT and S-K-BNT showed strong correlations with temporal regional volumes (Table 2; Figure 2).

**Table 2:** Correlations between frontal/executive functions and regional brain volume.

Brain regions			Frontal/executive functions				Attention		Language	
			COWAT	Stroop test colorreading	DSC	TMT_B	Digit span forward	Digit span backward	K-BNT	S-K-BNT
Temporal regions	Temporal anterior	Lt	0.316**	0.229*	0.221*	−0.230*	-	-	0.319**	0.311**
		Rt	0.267*	-	-	−0.271*	-	-	0.241*	0.328**
	Temporal medial	Lt	0.476**	0.318**	-	−0.364**	−0.218*	-	0.330**	0.343**
		Rt	0.406**	-	-	−0.337**	-	-	-	0.260*
	Temporal lateral	Lt	0.451**	0.293**	0.216*	−0.234*	-	-	0.400**	0.472**
		Rt	0.294**	-	-	-	-	-	-	0.345**
	Entorhinal cortex	Lt	0.380**	-	-	−0.235*	-	-	0.295**	0.312**
		Rt	0.339**	-	-	-	-	-	0.305**	0.308**
	Hippocampus	Lt	0.354**	-	-	-	−0.297**	-	0.353**	0.359**
		Rt	0.303**	-	-	-	−0.219*	-	0.295**	0.371**
Frontal regions	Orbitofrontal	Lt	0.327**	0.256*	-	-	-	-	-	-
		Rt	0.233*	-	-	-	-	-	-	-
	Frontal anterior	Lt	0.282**	-	-	−0.401**	-	-	-	-
		Rt	0.271*	-	-	-	-	0.218*	-	-
	Frontal anterior medial	Lt	-	-	-	-	-	-	-	-
		Rt	-	-	-	-	-	-	-	-
	Frontal dorsolateral	Lt	-	-	-	-	-	0.244*	-	-
		Rt	-	-	-	-	0.217*	0.288**	-	-
	Frontal inferior	Lt	0.216*	-	0.239*	−0.253*	-	-	-	-
		Rt	-	-	-	-	-	0.261*	-	-
	Frontal posterior medial	Lt	-	-	-	-	-	-	-	-
		Rt	-	-	-	-	-	-	-	-

*p < 0.05, **p < 0.005. Age, sex, and years of education were adjusted as confounding factors. Lt: Left orbitofrontal lobe; Rt: Right orbitofrontal lobe.

### 3.3 Memory functions exhibited various correlations with both the frontal and temporal lobes

As indicated by the results of the correlation analysis between memory function test scores and brain regions, both verbal learning & memory and visual learning & memory scores exhibited very strong correlations with all temporal regions. The correlation coefficients range from 0.217 to 0.581. Frontal region volumes also correlated with memory function, with correlation coefficients ranging from 0.215 to 0.449. With the exception of the RCFT recognition score, the other five memory function test scores exhibited some degree of correlation with the frontal regions, and the SVLT recognition score exhibited the strongest correlation between them.

**Figure 3:**
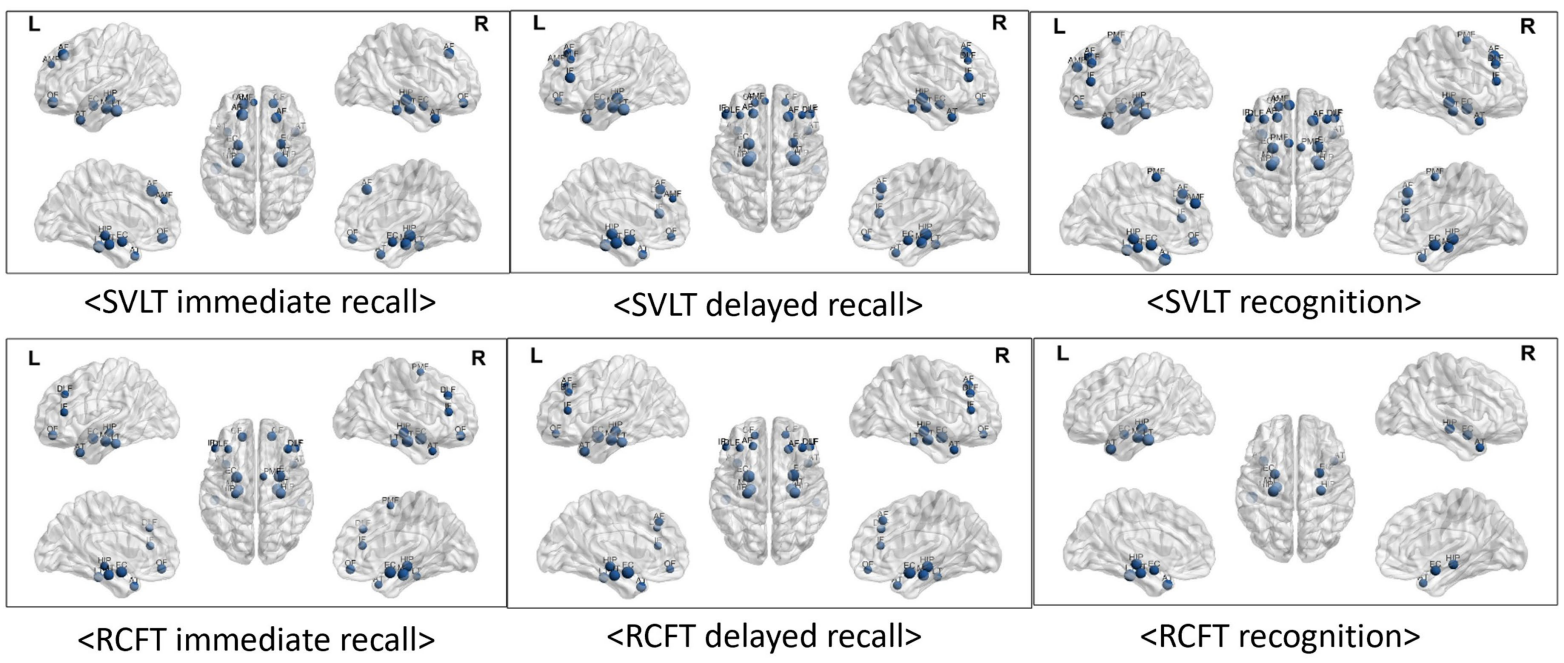
Visualized correlations between regional brain volume and memory function. Memory function tests exhibited extremely strong relationships with all of the temporal regions of the brain as observed by the results of the correlation study between test scores and brain regions. The results correlation coefficients varied from 0.216 to 0.581. A link between frontal area volumes and memory functions was also observed, with correlation values varying between 0.215 and 0.449. With the exception of the RCFT recognition score, the results of the other five memory function tests exhibited some relationships with frontal regions, and the SVLT recognition score exhibited the strongest correlations. Furthermore, there was a correlation between the RCFT copy score that represents visuospatial function and the inferior frontal (r = 0.239, *p* < 0.05), temporal anterior (r = 0.221, *p* < 0.05), and lateral regions (r = 0.216, *p* < 0.05).

Additionally, visuospatial function represented by the RCFT copy score was correlated with all temporal regions and both sides of orbitofrontal region (Table 3; Figure 3).

**Table 3:** Correlations between memory functions and regional brain volume.

Brain regions			Verbal learning & memory			Visual learning & memory			Visuospatial function
			SVLT immediate recall	SVLT delayed recall	SVLT recognition	RCFT immediate recall	RCFT delayed recall	RCFT recognition	RCFT copy score
Temporal regions	Temporal anterior	Lt	0.317**	0.349**	0.439**	0.300**	0.377**	0.415**	0.262*
		Rt	0.318**	0.315**	0.271*	0.241*	0.316**	0.396**	0.304**
	Temporal medial	Lt	0.387**	0.474**	0.338**	0.364**	0.445**	0.422**	0.241*
		Rt	0.422**	0.436**	0.339**	0.424**	0.442**	0.488**	0.280*
	Temporal lateral	Lt	0.452**	0.501**	0.406**	0.334**	0.402**	-	0.322**
		Rt	0.359**	0.347**	-	0.237*	0.303**	-	0.311**
	Entorhinal cortex	Lt	0.369**	0.477**	0.396**	0.391**	0.505**	0.526**	0.291**
		Rt	0.334**	0.373**	0.404**	0.425**	0.487**	0.581**	0.359**
	Hippocampus	Lt	0.359**	0.471**	0.397**	0.260*	0.390**	0.355**	0.307**
		Rt	0.445**	0.497**	0.419**	0.365**	0.444**	0.412**	0.282**
Frontal regions	Orbitofrontal	Lt	0.403**	0.281**	0.309**	0.306**	0.275*	-	0.230*
		Rt	0.334**	0.260*	-	0.311**	0.273*	-	0.261*
	Frontal anterior	Lt	0.449**	0.381**	0.336**	-	0.294**	-	-
		Rt	0.389**	0.418**	0.389**	-	0.324**	-	-
	Frontal anterior medial	Lt	0.217*	0.225*	0.365**	-	-	-	-
		Rt	-	-	-	-	-	-	-
	Frontal dorsolateral	Lt	-	0.270*	0.316**	0.245*	0.262*	-	-
		Rt	-	0.258*	0.292**	0.275*	0.321**	-	-
	Frontal inferior	Lt	-	0.404**	0.305**	0.251*	0.270*	-	-
		Rt	-	0.379**	0.287**	0.271*	0.287**	-	-
	Frontal posterior medial	Lt	-	-	0.284**	-	-	-	-
		Rt	-	-	0.253*	0.215*	-	-	-

*p < 0.05, **p < 0.005. Age, sex, and years of education were adjusted as confounding factors. Lt: Left orbitofrontal lobe; Rt: Right orbitofrontal lobe.

## 4 Discussion

We started this retrospective study of 93 confirmed AD patients to determine the correlation between cognitive function and quantitative brain volume. Here, we confirmed that (1) frontal function test results (COWAT, Stroop test, DSC and TMT) correlated significantly with temporal regions, but few significant correlation was observed with the frontal region itself. Also, (2) language function tests (K-BNT, S-K-BNT) showed strong correlations with temporal volume, but no significant correlations were observed with any of frontal volume. However, (3) memory function test results (Recognition, immediate and delayed recall of SVLT and RCFT) were correlated with both the frontal and temporal regions.

SVLT immediate recall evaluates verbal learning and is known to be related to the temporal lobe (Kang et al., 2019; Kang and Na, 2003). Our study results agree with this by demonstrating that SVLT immediate recall can exert a greater effect on the temporal lobe than it can on the frontal lobe. No left-sided dominance was observed. The SVLT delayed recall that involves inferring from hints and tactics and the SVLT recognition score correlated with both the temporal and frontal lobes. One study opposed the result that delayed recall can be more closely related to the regional temporal lobe than immediate recall (Kang et al., 2019). They indicated that immediate recall can include not only memory but also the involvement of other cognitive subfunctions such as learning.

However, correlations of RCFT recall scores revealed that visuospatial memory appears to possess widespread correlations beyond whole-brain regions (Meyers and Meyers, 1995). This is different from the finding that the RCFT recognition score correlated only with the temporal lobe. BNT which is used to evaluate naming and language function is known to be related to both the temporal and frontal regions (Putcha et al., 2020; Yeonwook et al., 1999). However, in our study of confirmed AD patients, it appears that only the temporal region is correlated with BNT scores. In addition, the frontal/executive function tests showed stronger correlations with temporal regions rather than frontal regions. Therefore, we suggest that the frontal lobe in patients with early AD should be evaluated using learning and memory rather than frontal/executive function test results.

Based on correlation analyses, we found that in patients with early-stage AD, frontal function exhibited a stronger correlation with the temporal atrophy rather than with the frontal region itself. This finding implies that monitoring non-amnestic MCI (Mild cognitive impairment) patients with temporal atrophy may provide valuable insights into the progression to AD. Therefore, we propose that careful tracking of subtle cognitive changes in these patients is essential for understanding the potential transition to AD. Furthermore, as we expect that the progression of AD and specific behavioral characteristics can differ between patients with temporal and frontal atrophy, it is important to observe the results of neuropsychological tests and MRI scans in detail in the early stages of the disease.

A limitation of this retrospective study is that MRI scans and cognitive function tests were not performed simultaneously. We believe this has been overcome to some extent by the study design that restricted the period between MRI scans and cognitive function tests to less than 1 year, with an average of 22.7 days and a median of 3 days. Second, all research participants were right handed. Further research should be conducted in larger groups with varying handedness. Third, we did not explicitly assess performance validity during cognitive tests, assuming participants put forth their best effort. Thus, various factors such as mood, anxiety, and fatigue could not be excluded. Lastly, although we conducted additional subgroup analyses stratified by CDR scores, the limited sample size in each subgroup reduced the statistical power to detect significant associations. Future prospective studies with larger and more diverse samples are warranted to validate and extend our findings.

Another important consideration is determining the relationship between disease onset, volumetric brain changes, and cognitive performance, particularly given the recent guideline proposing that biomarker changes can precede objective cognitive impairment. While our retrospective study was limited by the absence of longitudinal data from early AD patients, future prospective studies are needed to clarify the relationship between early biomarkers, cognitive assessments, and volumetric brain imaging throughout AD progression.

## Data Availability

The raw data supporting the conclusions of this article will be made available by the authors, without undue reservation.
