# Supplementary material for: Unravelling the interplay: brain regional atrophy and neuropsychological function in early Alzheimer’s disease
Source: Front Aging Neurosci. 2025 May 14;17:1508849. doi: 10.3389/fnagi.2025.1508849 (PMC12116575; doi:10.3389/fnagi.2025.1508849)
Supplement: Supplementary file 1 [file Data_Sheet_1.docx]

|  |  |  | **Frontal/executive functions** | | |  |  | **Attention** |  | **Language** | |
| --- | --- | --- | --- | --- | --- | --- | --- | --- | --- | --- | --- |
|  |  |  | COWAT | Stroop Test  Colorreading | DSC | | TMT_B | Digit span Forward | Digit span Backward | K-BNT | S-K-BNT |
|  |  |  |  |  |  | |  |  |  |  |  |
|  |  |  |  |  |  | |  |  |  |  |  |
|  | Temporal anterior | Lt | 0.250* | - | - | | -0.381** | - | - | - | - |
|  |  | Rt | - | - | - | | -0.381** | - | - | - | - |
|  | Temporal medial | Lt | 0.442** | 0.304* | - | | -0.519** | - | - | - | - |
|  |  | Rt | 0.376** | - | - | | -0.473** | - | - | - | - |
| **Temporal regions** | Temporal lateral | Lt | 0.401** | - | - | | -0.301* | - | - | 0.356** | 0.368** |
|  |  | Rt | 0.361** | - | - | | - | - | - | - | 0.300* |
|  | Entorhinal cortex | Lt | 0.380** | 0.352** |  | | -0.403** | - | - | - | - |
|  |  | Rt | 0.331** | - | - | | - | - | - | - | - |
|  | Hippocampus | Lt | 0.323** | 0.409** | - | | - | - | - | - | - |
|  |  | Rt | - | - | - | | - | - | - | - | - |
|  | Orbitofrontal | Lt | 0.265* | - | - | | - | - | - | - | - |
|  |  | Rt | - | - | - | | - | - | - | - | - |
|  | Frontal anterior | Lt | 0.320* | - | - | | -0.445** | - | - | - | - |
|  |  | Rt | 0.291* | - | - | | - | - | - | - | - |
|  | Frontal anterior medial | Lt | - | - | - | | - | - | - | - | - |
| **Frontal regions** |  | Rt | - | - | - | | - | - | - | - | - |
|  | Frontal dorsolateral | Lt | - | - | - | | - | 0.323** | - | - | - |
|  |  | Rt | - | - | - | | - | 0.361** | - | - | 0.296* |
|  | Frontal inferior | Lt | 0.304* | - | - | | -0.253* | 0.269* | - | - | - |
|  |  | Rt | - | - | - | | - | 0.351** | - | - | - |
|  | Frontal posterior medial | Lt | - | - | - | | - | - | - | - | - |
|  |  | Rt | - | - | - | | - | - | - | - | - |

Supplementary Table 1. Partial correlations between Frontal/executive functions and regional brain volume in patients with CDR score of 0.5 (*p<0.05, **p<0.005, ***p<0.001)

Age, sex, and years of education were adjusted as confounding factors.

|  |  |  | **Verbal learning & memory** | | | | **Visual learning & memory** | | | **Visuospatial function** | | |
| --- | --- | --- | --- | --- | --- | --- | --- | --- | --- | --- | --- | --- |
|  |  |  | SVLT immediate recall | SVLT delayed recall | SVLT recognition | RCFT immediate recall | | RCFT delayed recall | RCFT recognition | | RCFT copy score |  |
|  |  |  |  |  |  |  | |  |  | |  |  |
|  |  |  |  |  |  |  | |  |  | |  |  |
|  | Temporal anterior | Lt | 0.322** | 0.314* | 0.409** | - | | 0.317* | 0.360** | | 0.301* |  |
|  |  | Rt | 0.305* | 0.283* | - | - | | 0.266* | 0.369** | | 0.297* |  |
|  | Temporal medial | Lt | 0.385** | 0.506** | 0.274* | 0.365** | | 0.457** | 0.413** | | 0.366** |  |
|  |  | Rt | 0.363** | 0.404** | 0.250* | 0.395** | | 0.400** | 0.493** | | 0.370** |  |
| **Temporal regions** | Temporal lateral | Lt | 0.431** | 0.482** | 0.360** | 0.268* | | 0.337** | - | | 0.405** |  |
|  |  | Rt | 0.314* | 0.316* | - | - | | 0.249* | - | | 0.338** |  |
|  | Entorhinal cortex | Lt | 0.338** | 0.483** | 0.378** | 0.364** | | 0.515** | 0.514** | | 0.403** |  |
|  |  | Rt | - | 0.311* | 0.363** | 0.393** | | 0.455** | 0.560** | | 0.493** |  |
|  | Hippocampus | Lt | 0.369** | 0.499** | 0.314* | - | | 0.377** | 0.296* | | 0.390** |  |
|  |  | Rt | 0.411** | 0.472** | 0.324** | 0.294* | | 0.386** | 0.415** | | 0.380** |  |
|  | Orbitofrontal | Lt | 00.305* | - | - | - | | - | - | | - |  |
|  |  | Rt | - | - | - | - | | - | - | | - |  |
|  | Frontal anterior | Lt | 0.474** | 0.393** | 0.329** | 0.310* | | 0.288* | - | | - |  |
|  |  | Rt | 0.367** | 0.399** | 0.374** | 0.279* | | 0.276* | - | | - |  |
|  | Frontal anterior medial | Lt | - | - | 0.426** | - | | - | - | | - |  |
| **Frontal regions** |  | Rt | 0.267^*^ | 0.270^*^ | 0.352** | - | | - | - | | - |  |
|  | Frontal dorsolateral | Lt | - | - | 0.368** | - | | - | - | | - |  |
|  |  | Rt | - | - | 0.288* | 0.300^*^ | | 0.299^*^ | - | | - |  |
|  | Frontal inferior | Lt | 0.304^*^ | 0.468^**^ | 0.429** | 0.315^*^ | | 0.319^*^ | - | | - |  |
|  |  | Rt | - | 0.368^**^ | 0.306* | 0.278^*^ | | 0.261^*^ | - | | - |  |
|  | Frontal posterior medial | Lt | - | - | 0.288* | - | | - | - | | - |  |
|  |  | Rt | - | - | 0.282* | - | | - | - | | - |  |

Supplementary Table 2. Partial correlations between Memory functions and regional brain volume in patients with CDR score of 0.5 (*p<0.05, **p<0.005, ***p<0.001)

Age, sex, and years of education were adjusted as confounding factors.

|  |  |  | **Frontal/executive functions** | | |  |  | **Attention** |  | **Language** | |
| --- | --- | --- | --- | --- | --- | --- | --- | --- | --- | --- | --- |
|  |  |  | COWAT | Stroop Test  Colorreading | DSC | | TMT_B | Digit span Forward | Digit span Backward | K-BNT | S-K-BNT |
|  |  |  |  |  |  | |  |  |  |  |  |
|  |  |  |  |  |  | |  |  |  |  |  |
|  | Temporal anterior | Lt | - | - | - | | - | - | - | - | - |
|  |  | Rt | - | - | - | | - | - | - | - | - |
|  | Temporal medial | Lt | - | - | - | | - | -0.499* | - | 0.606** | 0.621** |
|  |  | Rt | - | - | - | | - | - | - | - | 0.573* |
| **Temporal regions** | Temporal lateral | Lt | 0.499* | - | - | | - | -0.618** | - | - | 0.544* |
|  |  | Rt | - | - | - | | - | - | - | - | - |
|  | Entorhinal cortex | Lt | - | - | - | | - | - | - | - | - |
|  |  | Rt | - | - | - | | - | - | - | - | - |
|  | Hippocampus | Lt | - | - | - | | - | -0.567* | - | - | 0.501* |
|  |  | Rt | - | - | - | | - | -0.569* | - | - | - |
|  | Orbitofrontal | Lt | - | 0.525* | - | | - | - | - | - | - |
|  |  | Rt | - | - | - | | - | - | - | - | - |
|  | Frontal anterior | Lt | - | - | - | | - | - | - | - | - |
|  |  | Rt | - | - | - | | - | - | - | - | - |
|  | Frontal anterior medial | Lt | - | - | - | | - | - | - | - | - |
| **Frontal regions** |  | Rt | - | - | - | | - | - | - | - | - |
|  | Frontal dorsolateral | Lt | - | - | - | | - | - | - | - | - |
|  |  | Rt | - | - | - | | - | - | - | - | - |
|  | Frontal inferior | Lt | - | - | - | | - | - | - | - | - |
|  |  | Rt | - | - | - | | - | - | - | - | - |
|  | Frontal posterior medial | Lt | - | - | - | | - | - | - | - | - |
|  |  | Rt | - | - | - | | - | - | - | - | - |

Supplementary Table 3. Partial correlations between Frontal/executive functions and regional brain volume in patients with CDR score of 1 (*p<0.05, **p<0.005, ***p<0.001)

Age, sex, and years of education were adjusted as confounding factors.

|  |  |  | **Verbal learning & memory** | | | | **Visual learning & memory** | | | **Visuospatial function** | | |
| --- | --- | --- | --- | --- | --- | --- | --- | --- | --- | --- | --- | --- |
|  |  |  | SVLT immediate recall | SVLT delayed recall | SVLT recognition | RCFT immediate recall | | RCFT delayed recall | RCFT recognition | | RCFT copy score |  |
|  |  |  |  |  |  |  | |  |  | |  |  |
|  |  |  |  |  |  |  | |  |  | |  |  |
|  | Temporal anterior | Lt | - | 0.547* | - | 0.491* | | 0.631** | - | | - |  |
|  |  | Rt | - | - | - | - | | - | - | | - |  |
|  | Temporal medial | Lt | - | - | - | - | | - | - | | - |  |
|  |  | Rt | - | - | - | - | | - | - | | - |  |
| **Temporal regions** | Temporal lateral | Lt | - | - | - | - | | - | - | | - |  |
|  |  | Rt | - | - | - | - | | - | - | | - |  |
|  | Entorhinal cortex | Lt | - | 0.605* | - | - | | 0.527* | 0.483* | | - |  |
|  |  | Rt | - | - | - | - | | - | 0.534* | | - |  |
|  | Hippocampus | Lt | - | - | - | - | | - | - | | - |  |
|  |  | Rt | - | - | - | - | | - | - | | - |  |
|  | Orbitofrontal | Lt | 0.499* | - | - | - | | - | - | | - |  |
|  |  | Rt | - | - | - | - | | -0.503* | - | | - |  |
|  | Frontal anterior | Lt | - | - | - | - | | - | - | | - |  |
|  |  | Rt | - | - | - | - | | - | - | | - |  |
|  | Frontal anterior medial | Lt | - | - | - | - | | - | - | | - |  |
| **Frontal regions** |  | Rt | - | - | - | - | | - | - | | - |  |
|  | Frontal dorsolateral | Lt | - | - | - | - | | - | - | | - |  |
|  |  | Rt | - | - | - | - | | - | - | | - |  |
|  | Frontal inferior | Lt | - | - | - | - | | - | - | | - |  |
|  |  | Rt | - | - | - | - | | - | - | | - |  |
|  | Frontal posterior medial | Lt | - | - | - | - | | - | - | | - |  |
|  |  | Rt | - | - | - | - | | - | - | | - |  |

Supplementary Table 4. Partial correlations between Memory functions and regional brain volume in patients with CDR score of 1 (*p<0.05, **p<0.005, ***p<0.001)

Age, sex, and years of education were adjusted as confounding factors.
